# Comparative analysis of ECG records depending on body position in domestic swine (*Sus scrofa domestica*)

**DOI:** 10.1186/s40813-022-00282-x

**Published:** 2022-09-19

**Authors:** Marta Kawicka, Maksymilian Lewicki, Piotr Frydrychowski, Marcin Michałek, Agnieszka Noszczyk-Nowak

**Affiliations:** 1 The Faculty of Veterinary Medicine, Wrocław University of Environmental and Life Sciences, 31 Norwida St., 50-357 Wrocław, Poland; 2 Department of Internal Medicine and Clinic of Diseases of Horses, Dogs and Cats, The Faculty of Veterinary Medicine, Wrocław University of Environmental and Life Sciences, 47 Grunwaldzki Square, 50-366 Wrocław, Poland

**Keywords:** Body position, ECG, Pig, Heart, Cardiology, Veterinary

## Abstract

**Background:**

Electrocardiography is a method widely applied in diagnosing abnormalities in the functioning of the heart muscle in veterinary medicine. It is a non-invasive and easy to perform test helpful in the general examination and a widely used patient monitoring method during anesthesia. Since the 1980s, pigs have become more and more popular companion animals. Moreover, the pig is a widely used model animal in biomedical research. Therefore, there is need to provide them with higher-quality veterinary services, also in emergency situations. It creates new challenges for veterinarians and the need to expand their knowledge of pigs’ treatment as pets. The aim of the planned experiment was to compare the ECG recordings made with two different body positions and determine if any differences occurred. Standard ECG in swine is performed under general anesthesia in the lying position on the left side, for this position of the body have been developed and reported standards in the literature. However, some procedures performed on swine require a different body position, for which there is less data in the literature.

**Methods:**

The study was carried out on 29 Polish landrace pigs weighing in the range of 33–44 kg. The tests were performed under general anesthesia with the same protocol for each animal, placing the animals first lying down on their right side, and then on their backs. The anesthesia protocol included medetomidine, midazolam, ketamine, and propofol. During the examination, ECG records were performed and analyzed in a 12-lead system with software support.

**Results:**

The results show significant differences in electrocardiogram recordings depending on the animal's body position. Those differences mainly concern the amplitude of the P wave and R wave in the recordings and are even more visible comparing the electrocardiograms of the same specimen. There are also some significant differences in the duration of intervals. Based on the obtained results, reference ranges for the right lateral and dorsal positions were developed.

**Conclusion:**

In conclusion, the body position has a significant impact on the ECG recording in swine, therefore performing this examination, chosen normative value tables should be compatible with the position of the examined animal.

## Background

In North America, miniature pigs have increasingly become companion animals since the 1980s. When seeking medical advice, pig owners often seek the advice of a local small animal doctor [1, 2]. As with other companion animals, pigs may also require a dorsal position in surgical procedures such as an ovariohysterectomy. To ensure the safety of anesthesia during the procedure, intraoperative monitoring is necessary to assess the vital functions of the animal. Its important element is single-lead or multi-lead ECG examination, in which we can observe cardiac arrhythmias. The pig is a species different from that of dogs or cats. Therefore, the norms of physiological parameters for this species should be used during intraoperative monitoring, including the norms for electrocardiographic recordings [2]. Also, in emergencies, the companion pigs do not receive adequate medical treatment due to the limited knowledge and experience of veterinarians in the treatment of this species, which leads to increased mortality. This situation provokes veterinarians to constantly improve their knowledge of pigs’ diagnosis and treatment as pets [3].

Moreover, swine of different breeds and types are widely used as model animals in biomedical research to understand the cause, nature, and treatment options for human diseases [4–6]. Their high analogies in the structure of the cardiovascular system, such as the size of the vessels and heart cavities, heart rate, and physiological reactions as well as other systems, make swine excellent model animals for researchers [5–7]. Despite some differences in the construction of both the heart itself and the conductive system of pigs’ heart in comparison to humans, it is a useful object in model studies, including electrophysiology [8]. The pig is also a leading species under investigation for xenotransplantation between animals and humans [9–11]. Recent studies report on successful kidney and heart xenografts [12]. The swine is an animal well discovered not only as a laboratory animal but also as a farm animal, thanks to which a wide knowledge of its needs, specific maintenance, and welfare is available [13, 14]. Its breed dependent weight gain and time of sexual maturation allow for the quick and precise achievement of target parameters for research [6, 15]. Moreover, the pig as species is less controversial, relatively cheap, and widely available animal in comparison with monkeys [16]. Those factors, and a more similar structure of the coronary vessels than in dogs, make swine considerably better model animal [6].

The anatomy of the heart and structure of the conducting system in pigs has already been described, however, there is still little data on electrocardiography [7, 8, 17, 18]. One of the better-known breeds in terms of cardiovascular model studies is the Göttingen minipig [19, 20]. There are fewer cardiovascular studies in large breed swine model research. An important fact is the similarity of the coronary vessels in pigs and humans with the dominance of the right coronary artery. To compare, the dog's coronary vascular system varies more between individuals and the left coronary artery is dominant [5, 17]. In comparison to humans, swine’s sinoatrial node of conductive system lies lower on the septal wall and has different morphology [5]. Also, in swine bifurcation of His bundle is more proximal than in humans. By comparison, in dogs, the intraventricular pathway is trifascicular [5, 21]. A characteristic feature of pig and sheep hearts is the simultaneous depolarization within the epicardium and endocardium, it is suspected that a different structure of Purkinje fibers is responsible for this fact [5].

Standard ECG in these animals is performed under general anesthesia in the lying position on the left side. For this position of the body have been developed and reported standards in the literature [22]. However, some procedures performed on swine such as surgery and cardiovascular procedures, require a different body position, for which there is little data in the literature. Procedures where sternotomy is performed, such as cryoablation model studies, also require the animal to be placed on its back [23]. Although 12-lead ECGs were performed in some studies, there are no reference values nor normalized electrode placement system for 12-lead ECG in swine—human electrode placement system was used in the mentioned study [24]. There was also some research on ECG in swine where animals were not under anesthesia and were suspended in a harness keeping them in a standing position [25, 26]. Studies performed on dogs and humans show significant differences in ECG recordings dependent on body position changes [27, 28]. The study performed on dogs also suggested that using incorrect reference values for body position may lead to misinterpreting electrocardiograms [28].

The aim of the planned experiment was to observe and compare ECG recordings made with two different body positions and determine if any differences occurred. If there are any significant differences in ECG recordings, normative values for the dorsal and right lateral body position will be prepared.

## Results

All examined animals showed sinus rhythm.

### Heart rate

**Fig. 1:**
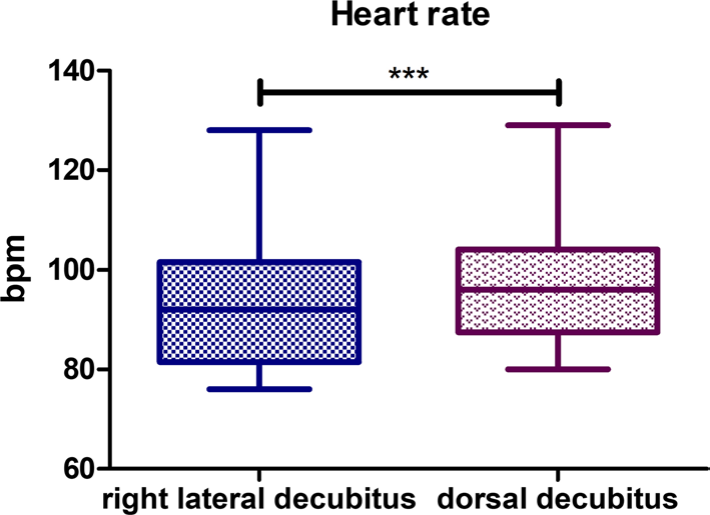
Comparison of heart rate (beats per minute–bpm) in right lateral and dorsal recumbency. T test for dependent pairs performed on 29 animals, where the values in the same specimen obtained in 2 body positions constitute a pair. Box plot contains 25% to 75% percentile, the inside line represents median, and whiskers represent minimal and maximal values (***P < 0,001)

Statistically, a significant difference was observed for the heart rate parameter (Fig. 1).

### Electrical heart axis

**Fig. 2:**
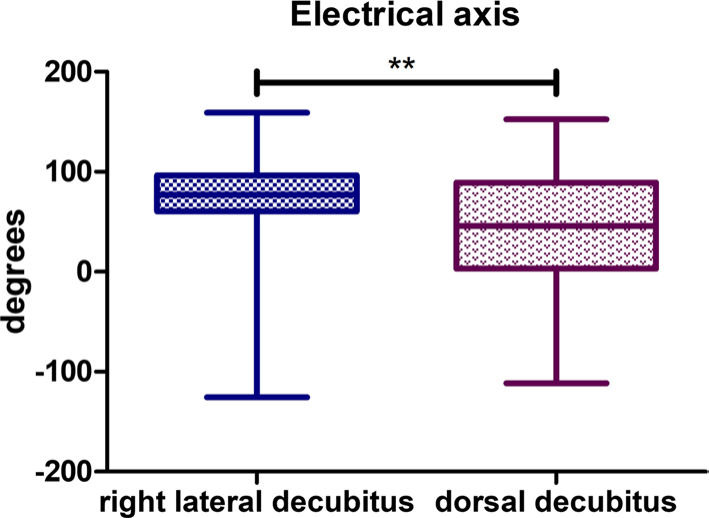
Comparison of electrical axis in degrees in both body positions. T test for dependent pairs performed on 29 animals, where the values in the same specimen obtained in 2 body positions constitute a pair. Box plot contains 25% to 75% percentile, the inside line represents median, and whiskers represent minimal and maximal values (**P < 0,01)

For the lateral position, a normal heart axis was observed in 19 animals, in 9 animals, a right deviation was observed. One animal in lateral position had extreme heart axis deviation. A normal heart axis was also observed in 19 animals in the dorsal body position. However, these are not the results of the same animals. Right axis deviation was observed in 6 animals, and left axis deviation was observed in 2 animals. One animal in dorsal position had extreme heart axis deviation. There was also one animal that resulted with a heart axis on the border between right deviation and extreme heart axis deviation. The mean electrical axis of the heart in the lateral position was 72.37 degrees, while in the dorsal position it was 43.86 degrees (*P*-value = 0,0067) (Fig. 2).

### Lead I

**Fig. 3:**
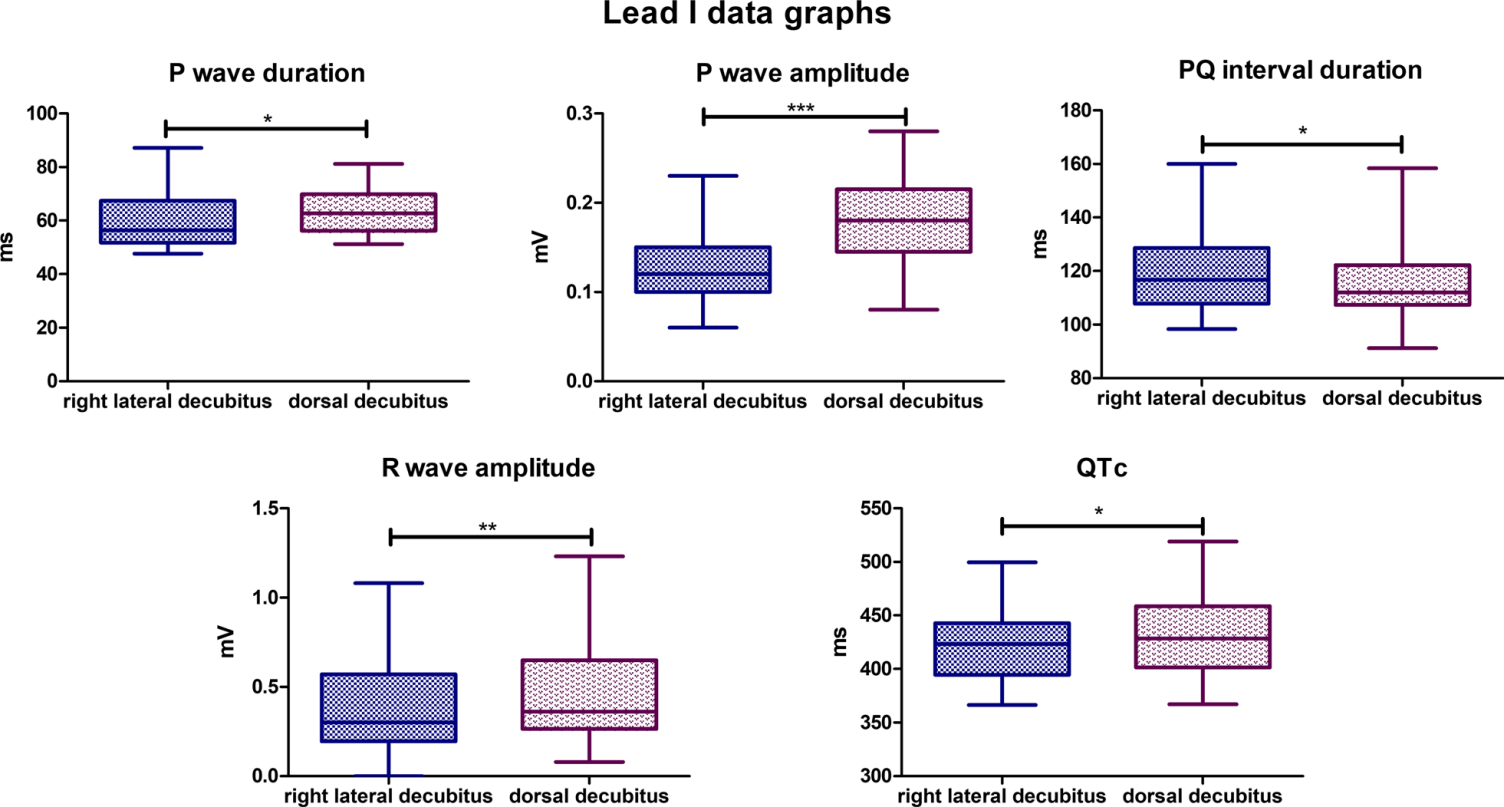
Comparison of the recording parameters differing statistically significantly in lead I. T test for dependent pairs performed on 29 animals, where the values in the same specimen obtained in 2 body positions constitute a pair. Box plot contains 25% to 75% percentile, the inside line represents median, and whiskers represent minimal and maximal values (*P < 0,05, **P < 0,01, ***P < 0,001)

In visual evaluation, the recording in the dorsal position is characterized by a greater amplitude compared to the lateral position. Statistically significant differences were observed for the following parameters. The mean P wave duration for the lateral body position was 60,17 ms, for the dorsal body position, the mean value was slightly higher and was 63,28 ms (*P*-value = 0,0353). The mean P wave amplitude value for the lateral body position was 0,1231 mV, while for the dorsal position, it was 0,1783 mV (*P*-value < 0,0001). The mean PQ interval duration value for lateral body position was 119,1 ms, while for the dorsal position, it was 115,4 ms (*P*-value = 0,0284). The mean R wave amplitude for the lateral body position was 0,3734 mV, and for the dorsal body position, the mean value was 0,4759 mV (*P*-value = 0,0057). The mean value for corrected QT Interval in lateral position was 422,6 ms, while for the dorsal position, it was 429,7 ms (*P*-value = 0,0102). Biphasic P wave was observed in 7 specimens in dorsal body position, negative deflection varied from − 0,02 to − 0,11 mV. In lateral position, biphasic P wave was observed in 3 animals and negative deflection varied from − 0,02 to − 0,08 mV (Fig. 3). In lateral position absence of R wave was observed in 1 specimen. No statistically significant differences were observed for the other parameters tested in lead I.

### Lead II

**Fig. 4:**
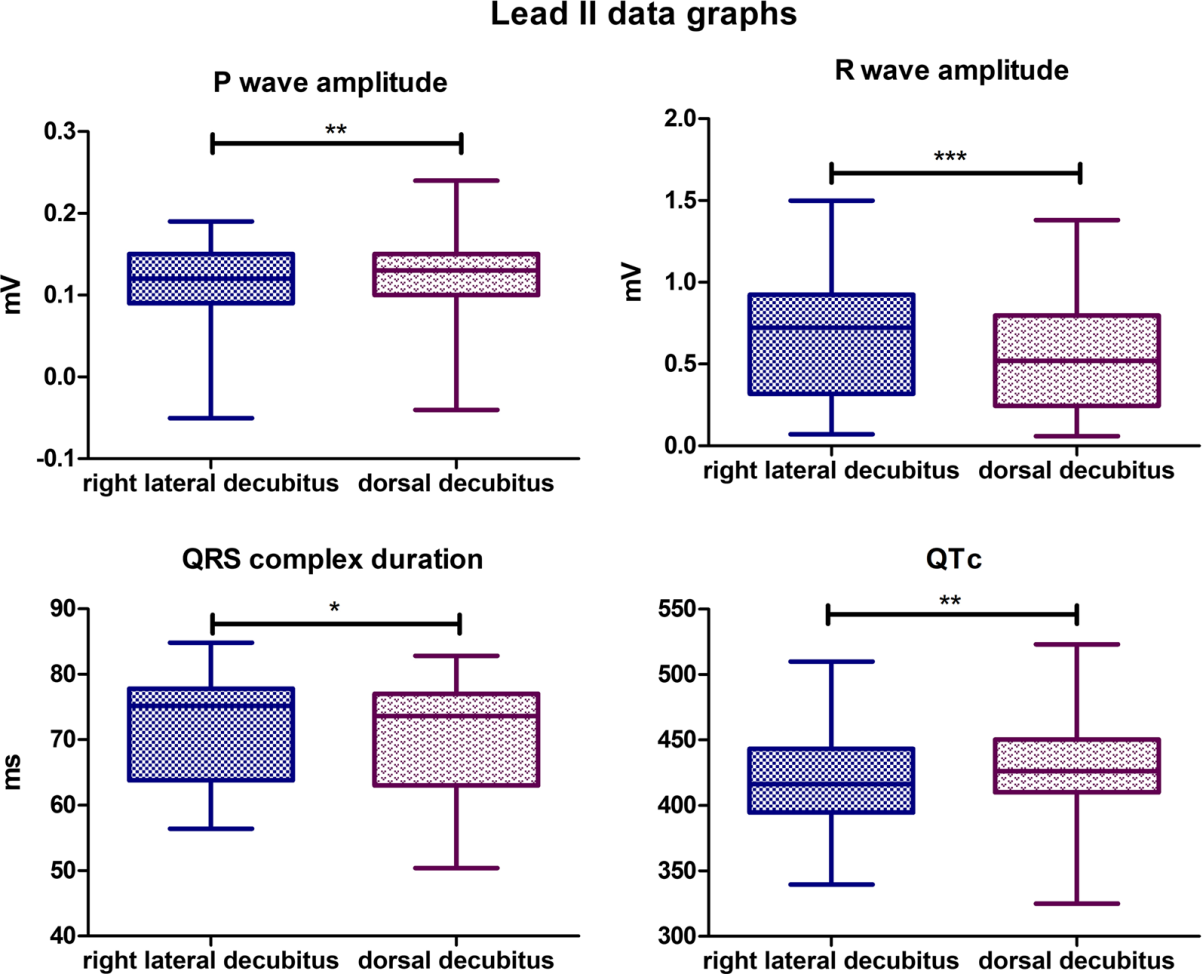
Comparison of the recording parameters differing statistically significantly in lead II. T test for dependent pairs performed on 29 animals, where the values in the same specimen obtained in 2 body positions constitute a pair. Box plot contains 25% to 75% percentile, the inside line represents median, and whiskers represent minimal and maximal values (*P < 0,05, **P < 0,01, ***P < 0,001)

In visual inspection, similar to lead I, the recording is characterized by a higher amplitude in the dorsal body position. Statistically significant differences were found for the following parameters. The mean P wave amplitude value for the lateral body position was 0,1131 mV, while for the dorsal position, it was 0,1290 mV (*P*-value = 0,0064). The mean R wave amplitude for the lateral body position was 0,6662 mV, and for the dorsal body position, the mean value was 0,5545 mV (*P*-value = 0,0003). The mean QRS complex duration for the lateral body position was 72,73 ms, for the dorsal body position, the mean value was lower and was 70,98 ms (*P*-value = 0,0392). The mean value for corrected QT Interval in lateral position was 421,4 ms, while for the dorsal position, it was 428,9 ms (*P*-value = 0,0077) (Fig. 4). Biphasic P wave was observed in 10 specimens in dorsal body position, negative deflection varied from − 0,08 to − 0,01 mV. In lateral position, biphasic P wave was observed in 11 animals, and negative deflection varied from − 0,02 to − 0,07 mV. R’ wave was observed in 7 specimens in lateral position with amplitude varying from 0,05 to 0,24 mV. In dorsal body position R’ wave was observed in 6 animals with amplitude varying from 0,06 to 0,19 mV. No statistically significant differences were observed for the other parameters tested in lead II.

### Lead III

**Fig. 5:**
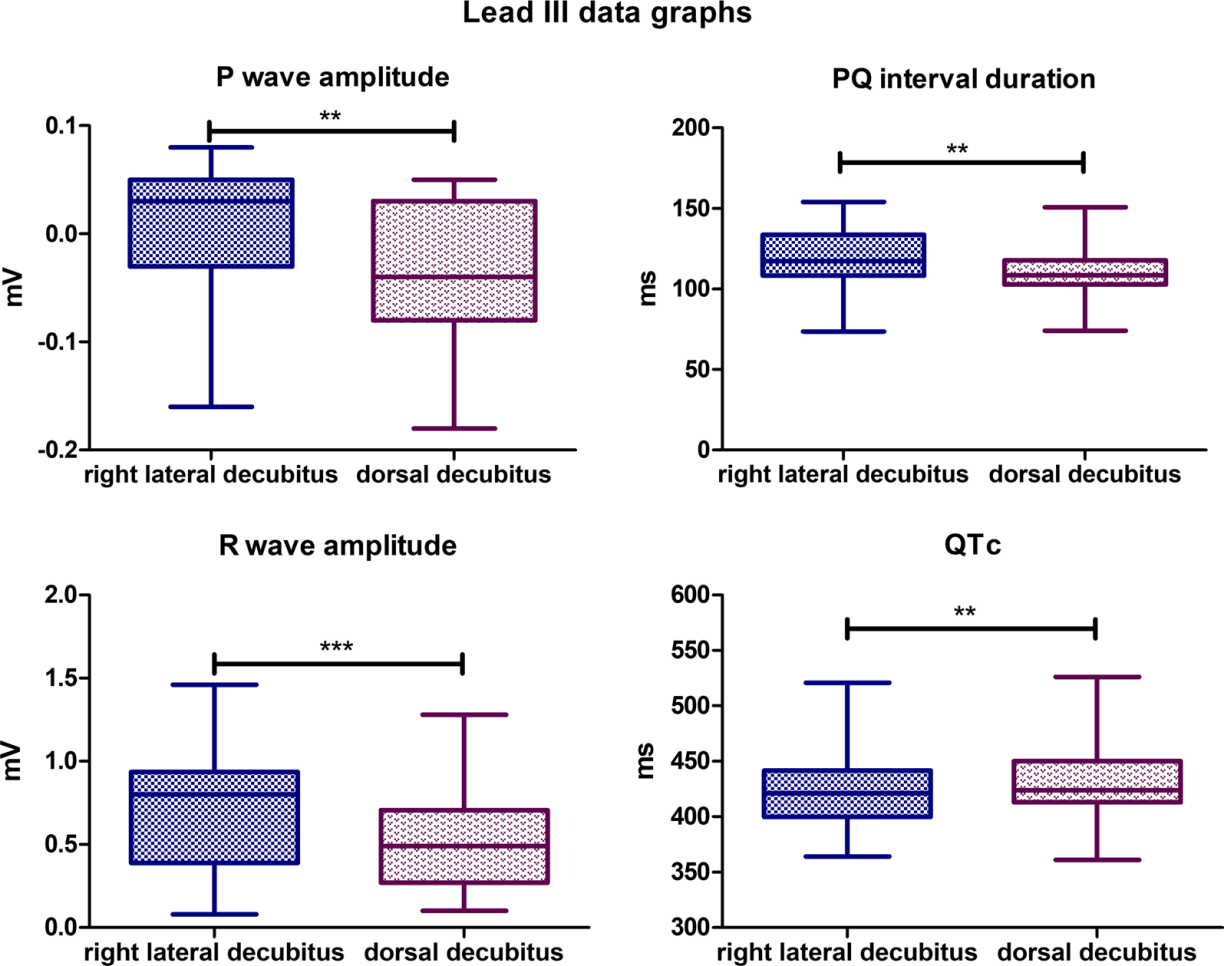
Comparison of the recording parameters differing statistically significantly in lead III. T test for dependent pairs performed on 29 animals, where the values in the same specimen obtained in 2 body positions constitute a pair. Box plot contains 25% to 75% percentile, the inside line represents median, and whiskers represent minimal and maximal values (**P < 0,01, ***P < 0,001)

In visual evaluation, the recording in the lateral position is characterized by a greater amplitude compared to the dorsal position. Statistically significant differences were observed for the following parameters. The mean P wave amplitude value for the lateral body position was 0,0031 mV, while for the dorsal position, it was − 0,0307 mV (*P*-value = 0,0011). The mean PQ interval duration value for lateral body position was 119,5 ms, while for the dorsal position, it was 109,9 ms (*P*-value = 0,0035). The mean R wave amplitude for the lateral body position was 0,7148 mV, and for the dorsal body position, the mean value was 0,5114 mV (*P*-value < 0,0001). The mean value for corrected QT Interval in lateral position was 422,6 ms, while for the dorsal position it was 430,5 ms (*P*-value = 0,0042) (Fig. 5). Biphasic P wave was observed in 6 specimens in dorsal body position, negative deflection varied from − 0,02 to − 0,08 mV. In lateral position, biphasic P wave was observed in 4 animals, and negative deflection also varied from − 0,02 to − 0,08 mV. R’ wave was observed in 4 specimens in lateral position with amplitude varying from 0,03 to 0,19 mV. In dorsal body position R’ wave was observed in 3 animals with amplitude varying from 0,03 to 0,07 mV. No statistically significant differences were observed for the other parameters tested in lead III.

### Lead aVR

**Fig. 6:**
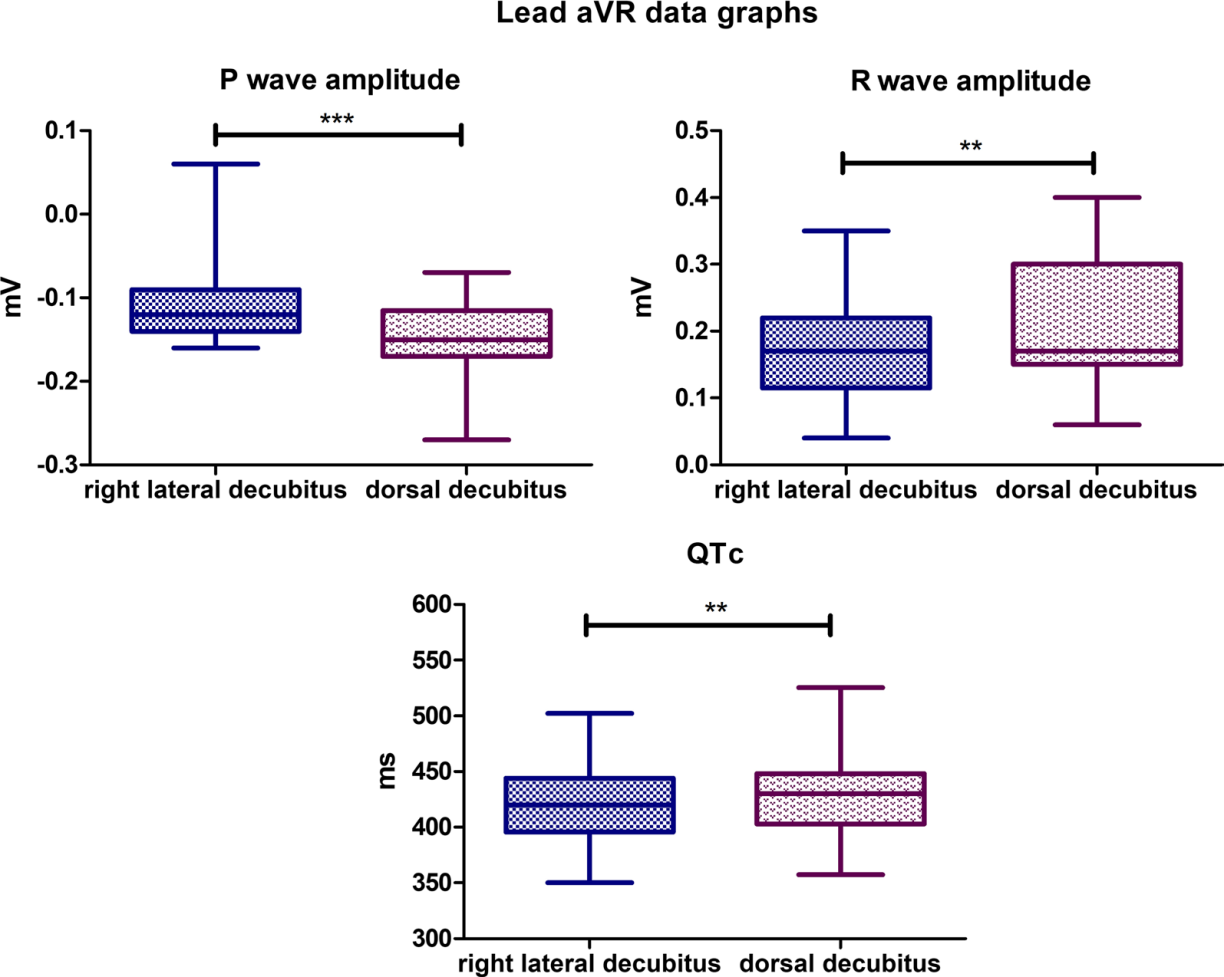
Comparison of the recording parameters differing statistically significantly in lead aVR. T test for dependent pairs performed on 29 animals, where the values in the same specimen obtained in 2 body positions constitute a pair. Box plot contains 25% to 75% percentile, the inside line represents median, and whiskers represent minimal and maximal values (**P < 0,01, ***P < 0,001)

Statistically significant differences were observed for the following parameters. The mean P wave amplitude value for the lateral body position was − 0,1117 mV, while for the dorsal position, it was − 0,1493 mV (*P*-value < 0,0001). The mean R wave amplitude for the lateral body position was 0,1728 mV, and for the dorsal body position, the mean value was 0,2117 mV (*P*-value = 0,0019). The mean value for corrected QT Interval in lateral position was 420,3 ms, while for the dorsal position, it was 428,4 ms (*P*-value = 0,0070) (Fig. 6). Biphasic P wave was observed in 9 specimens in dorsal body position, positive deflection varied from 0,02 to 0,1 mV. In lateral position, biphasic P wave was observed in 6 animals, and deflection varied from 0,01 to 0,07 mV. In dorsal body position R’ wave was observed in 5 animals with amplitude varying from 0,02 to 0,43 mV. R’ wave was observed in 3 specimens in lateral position with amplitude varying from 0,08 to 0,21 mV. No statistically significant differences were observed for the other parameters tested in lead aVR.

### Lead aVL

**Fig. 7:**
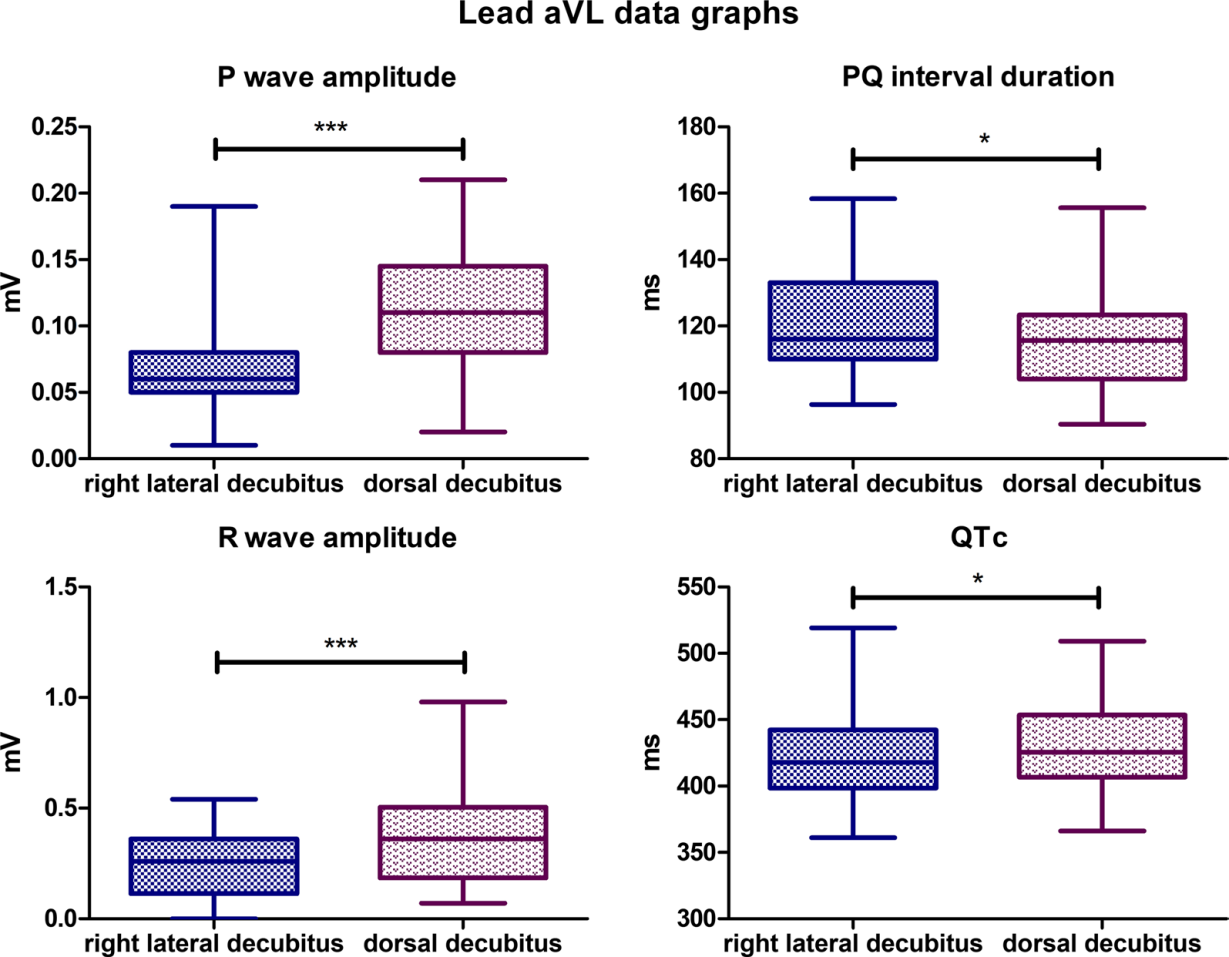
Comparison of the recording parameters differing statistically significantly in lead aVL. T test for dependent pairs performed on 29 animals, where the values in the same specimen obtained in 2 body positions constitute a pair. Box plot contains 25% to 75% percentile, the inside line represents median, and whiskers represent minimal and maximal values (*P < 0,05, ***P < 0,001)

The mean P wave amplitude value for the lateral body position was 0,06,793 mV, while for the dorsal position it was 0,1121 mV (*P*-value < 0,0001). The mean PQ interval duration value for lateral body position was 120,6 ms, while for the dorsal position it was 115,6 ms (*P*-value = 0,0160). The mean R wave amplitude for the lateral body position was 0,2472 mV, and for the dorsal body position, the mean value was 0,3876 mV (*P*-value = 0,0006). The mean value for corrected QT Interval in lateral position was 421,2 ms, while for the dorsal position, it was 427,9 ms (*P*-value = 0,0120) (Fig. 7). Biphasic P wave was observed in 6 specimens in dorsal body position, negative deflection varied from − 0,02 to − 0,08 mV. In lateral position, biphasic P wave was observed in 2 animals, and negative deflection varied from − 0,03 to − 0,05 mV. R’ wave was observed in 2 specimens in dorsal position with amplitude varying from 0,04 to 0,05 mV. In lateral body position R’ wave was observed in 10 animals with amplitude varying from 0,03 to 0,15 mV. Also, in lateral body position absence of R wave was observed in 4 animals. No statistically significant differences were observed for the other parameters tested in lead aVL.

### Lead aVF

**Fig. 8:**
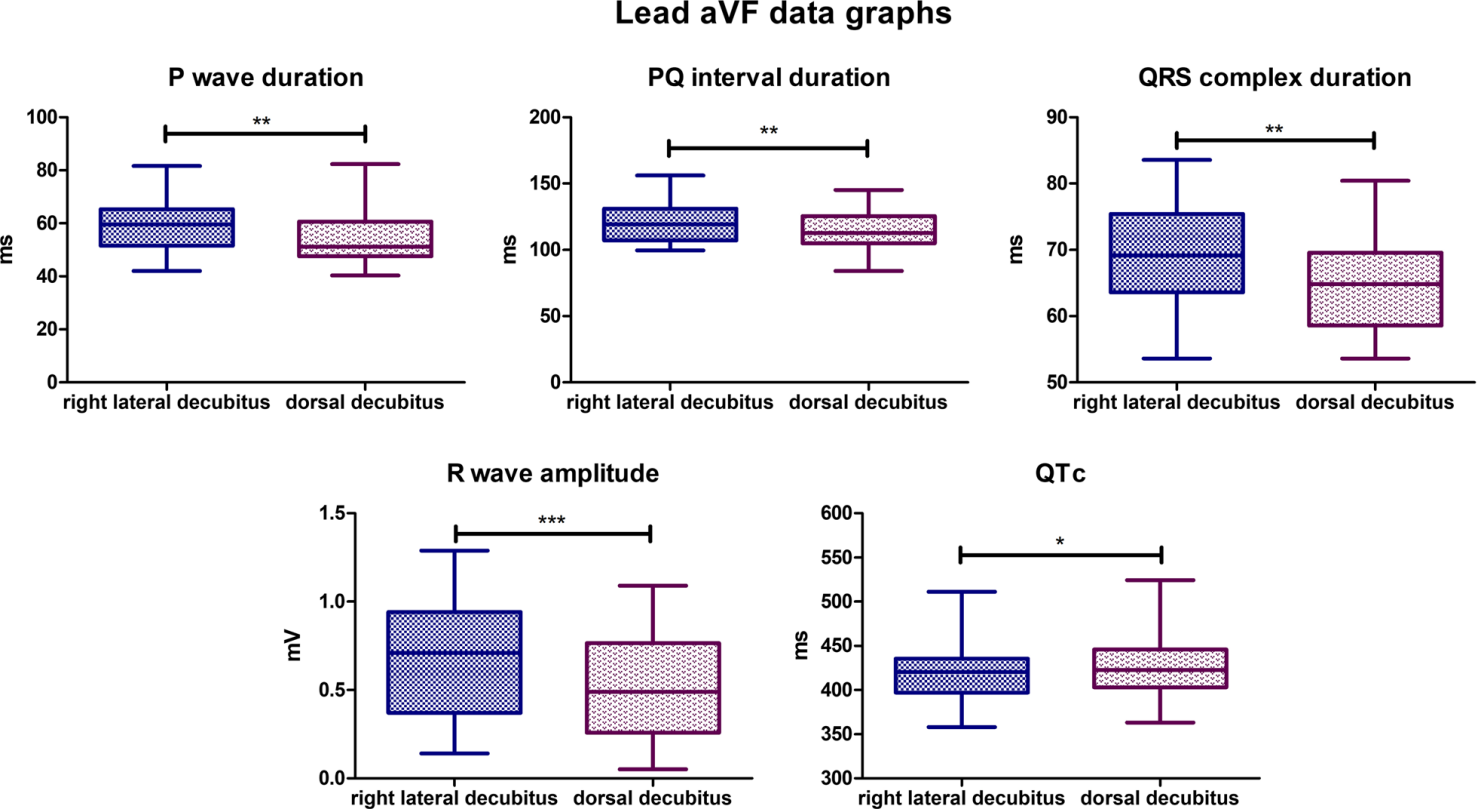
Comparison of the recording parameters differing statistically significantly in lead aVF. T test for dependent pairs performed on 29 animals, where the values in the same specimen obtained in 2 body positions constitute a pair. Box plot contains 25% to 75% percentile, the inside line represents median, and whiskers represent minimal and maximal values (*P < 0,05, **P < 0,01)

Statistically significant differences were observed for 5 parameters of the recording. The mean P wave duration for the lateral body position was 59,49 ms, for the dorsal body position, the mean value was slightly lower and was 54,15 ms (*P*-value = 0,0019). The mean PQ interval duration value for lateral body position was 121,2 ms, while for the dorsal position it was 115,3 ms (*P*-value = 0,0030). The mean QRS complex duration for the lateral body position was 69,35 ms, for the dorsal body position the mean value was lower and was 64,30 ms (*P*-value = 0,0041). The mean R wave amplitude for the lateral body position was 0,6807 mV, and for the dorsal body position, the mean value was 0,4941 mV (*P*-value < 0,0001). The mean value for corrected QT Interval in lateral position was 421,9 ms, while for the dorsal position, it was 427,8 ms (*P*-value = 0,0248) (Fig. 8). Biphasic P wave was observed in 7 specimens in lateral body position, negative deflection varied from − 0,02 to − 0,06 mV. In dorsal position, biphasic P wave was observed in 2 animals and negative deflection varied from − 0,01 to − 0,03 mV. In dorsal body position R’ wave was observed in 4 animals with amplitude varying from 0,03 to 0,1 mV. R’ wave was observed in 5 specimens in lateral position with amplitude varying from 0,03 to 0,11 mV. No statistically significant differences were observed for the other parameters tested in lead aVF.

### Lead V1

**Fig. 9:**
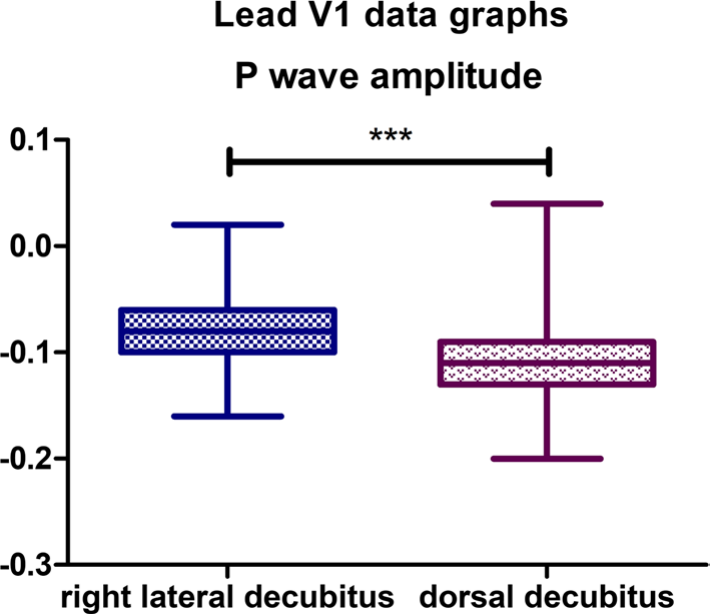
Comparison of the recording parameters differing statistically significantly in lead V1. T test for dependent pairs performed on 29 animals, where the values in the same specimen obtained in 2 body positions constitute a pair. Box plot contains 25% to 75% percentile, the inside line represents median, and whiskers represent minimal and maximal values (***P < 0,001)

Statistically significant differences were observed for only one parameter. The mean P wave amplitude value for the lateral body position was − 0,07,966 mV, while for the dorsal position it was − 0,1066 mV (*P*-value < 0,0001) (Fig. 9). Biphasic P wave was observed in 2 specimens in lateral body position, positive deflection varied from 0,02 to 0,03 mV. In dorsal position, biphasic P wave was observed in 4 animals, and positive deflection varied from 0,03 to 0,07 mV. In dorsal body position, R’ wave was observed in 17 animals with amplitude varying from 0,02 to 0,14 mV. R’ wave was observed in 12 specimens in lateral position with amplitude varying from 0,02 to 0,12 mV. No other statistically significant differences were observed in lead V1.

### Lead V2

**Fig. 10:**
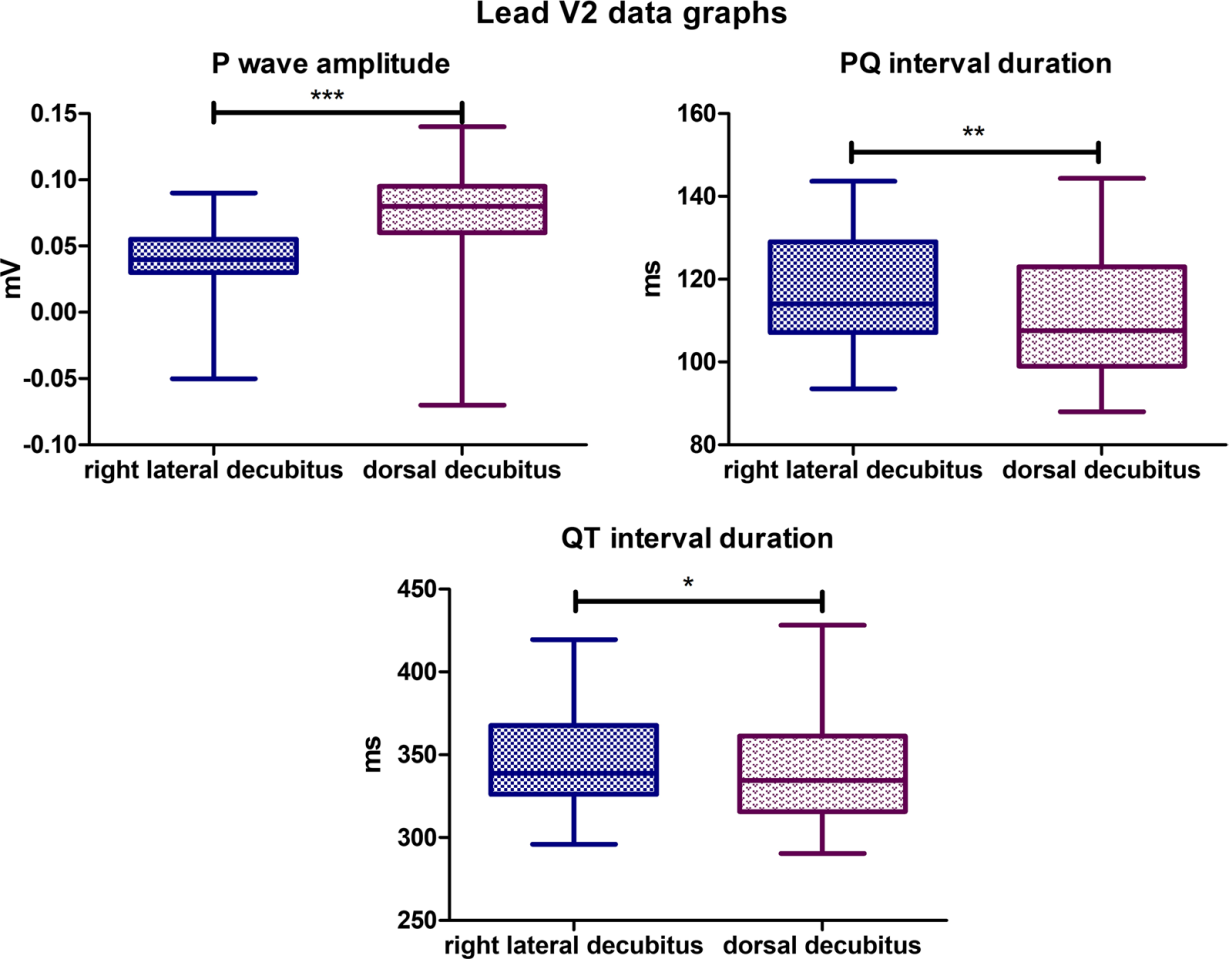
Comparison of the recording parameters differing statistically significantly in lead V2. T test for dependent pairs performed on 29 animals, where the values in the same specimen obtained in 2 body positions constitute a pair. Box plot contains 25% to 75% percentile, the inside line represents median, and whiskers represent minimal and maximal values (*P < 0,05, **P < 0,01, ***P < 0,001)

The mean P wave amplitude value for the lateral body position was 0,04,172 mV, while for the dorsal position, it was 0,07,276 mV (*P*-value < 0,0001). The mean PQ interval duration value for lateral body position was 117,2 ms, while for the dorsal position it was 111,9 ms (*P*-value = 0,0021). The mean QT Interval duration in the lateral position was 346,4 ms, while for the dorsal position, it was 342,0 ms (*P*-value = 0,0262) (Fig. 10). In both lateral and dorsal body positions, absence of R wave was observed in 4 specimens. Also, in dorsal body position presence of R’ wave was observed in 3 specimens with amplitude varying between 0,02 and 0,03 mV. No R’ waves were observed in lateral body position. No statistically significant differences were observed for the other parameters tested in lead V2.

### Lead V3

**Fig. 11:**
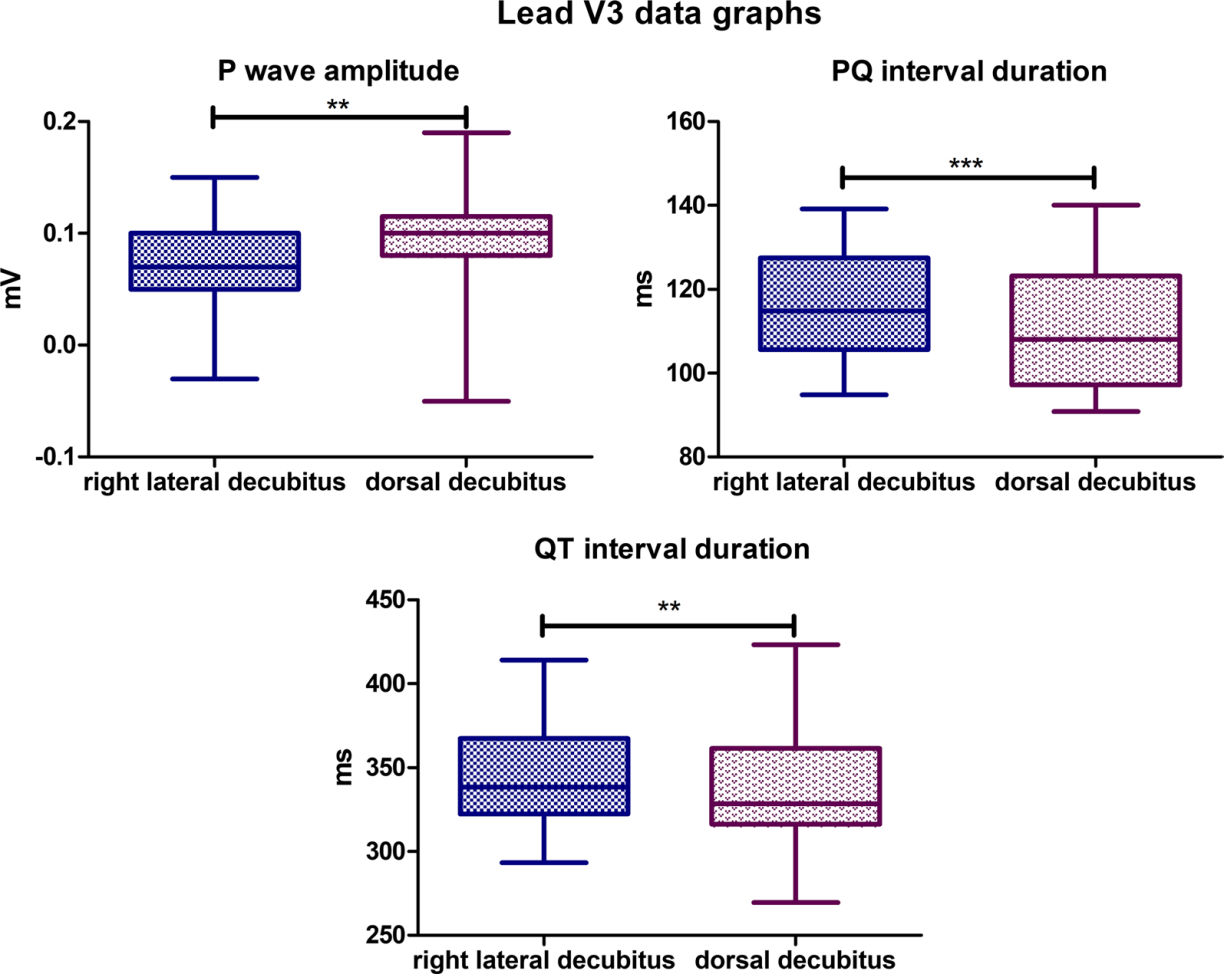
Comparison of the recording parameters differing statistically significantly in lead V3. T test for dependent pairs performed on 29 animals, where the values in the same specimen obtained in 2 body positions constitute a pair. Box plot contains 25% to 75% percentile, the inside line represents median, and whiskers represent minimal and maximal values (**P < 0,01, ***P < 0,001)

The mean P wave amplitude value for the lateral body position was 0,07,448 mV, while for the dorsal position, it was 0,09,483 mV (*P*-value = 0,0025). The mean PQ interval duration value for lateral body position was 116,5 ms, while for the dorsal position it was 111,2 ms (*P*-value = 0,0007). The mean QT Interval duration in the lateral position was 345,0 ms, while for the dorsal position it was 338,1 ms (*P*-value = 0,0045) (Fig. 11). In lateral body position, the absence of R wave was observed in 5 specimens. In dorsal position, 6 animals did not have R waves in a recording. No biphasic P waves nor R’ waves were observed in both positions. No statistically significant differences were observed for the other parameters tested in lead V3.

### Lead V4

**Fig. 12:**
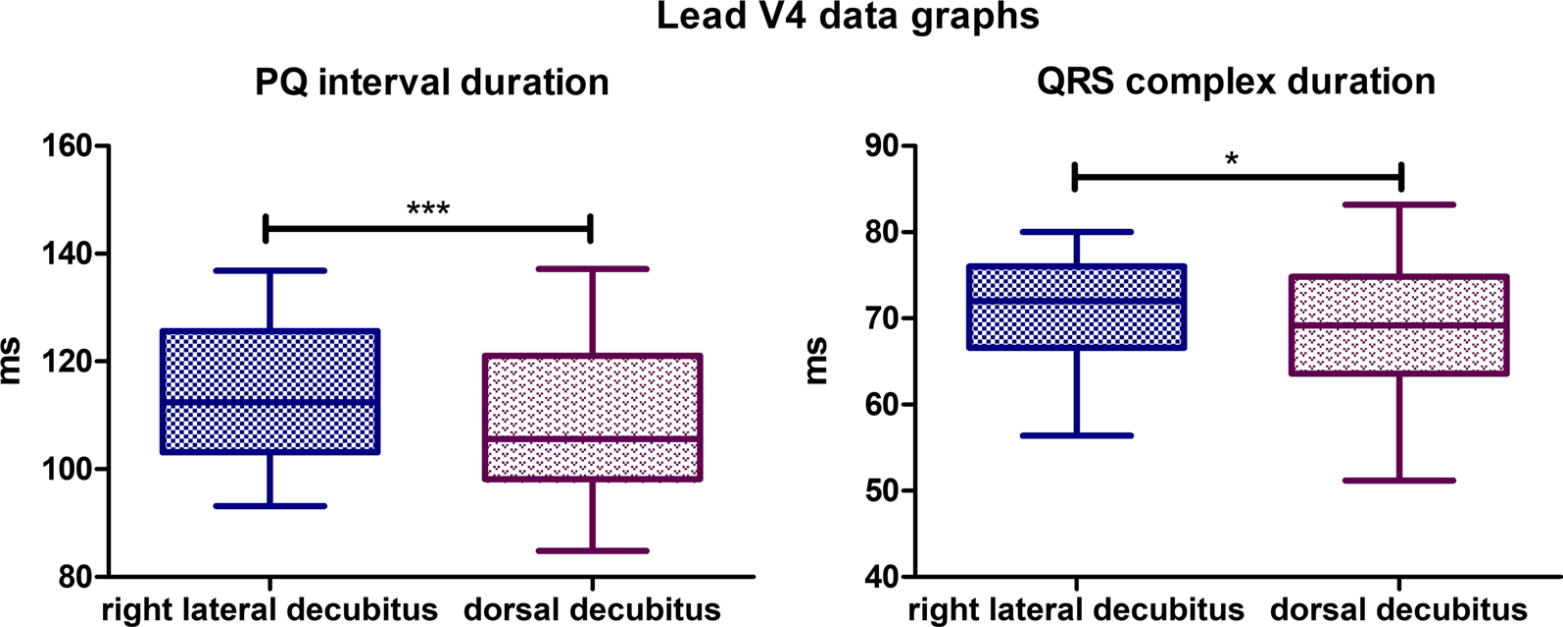
Comparison of the recording parameters differing statistically significantly in lead V4. T test for dependent pairs performed on 29 animals, where the values in the same specimen obtained in 2 body positions constitute a pair. Box plot contains 25% to 75% percentile, the inside line represents median, and whiskers represent minimal and maximal values (*P < 0,05, ***P < 0,001)

Statistically significant differences were observed for 2 parameters of the recording in lead V4. The mean PQ interval duration value for lateral body position was 114,5 ms, while for the dorsal position, it was 109,2 ms (*P*-value < 0,0001). The mean QRS complex duration for the lateral body position was 71,17 ms, for the dorsal body position the mean value was lower and was 68,58 ms (*P*-value = 0,0246) (Fig. 12). In lateral, no biphasic P wave nor R’ wave was observed. In dorsal body position in 1 specimen R’ wave was observed with amplitude varying from 0,05 to 0,11 mV. The absence of R wave was observed in 8 animals in dorsal body position, while in lateral body position, the absence of R wave was observed in 5 specimens.

### Lead V5

**Fig. 13:**
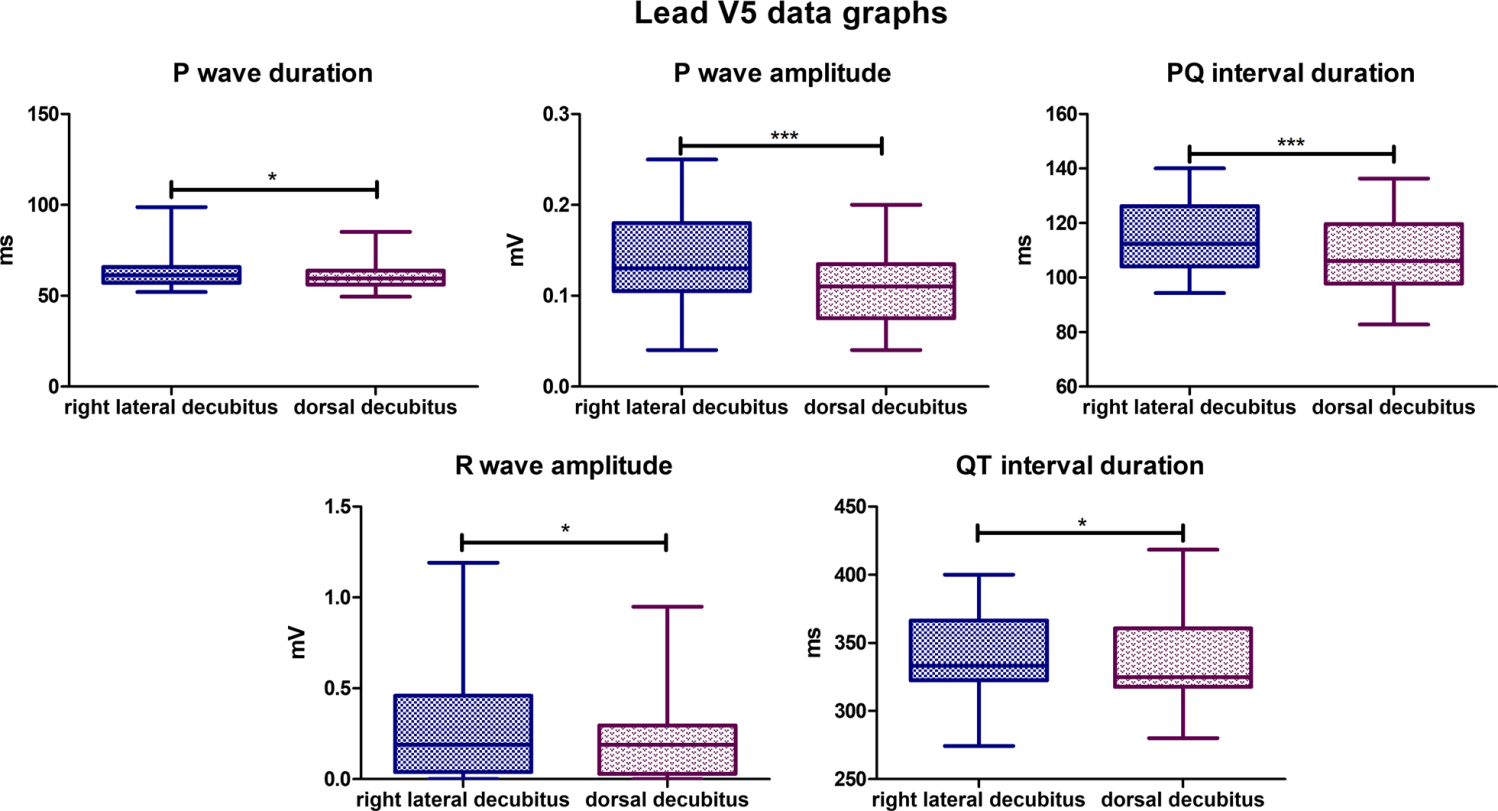
Comparison of the recording parameters differing statistically significantly in lead V5. T test for dependent pairs performed on 29 animals, where the values in the same specimen obtained in 2 body positions constitute a pair. Box plot contains 25% to 75% percentile, the inside line represents median, and whiskers represent minimal and maximal values (*P < 0,05, ***P < 0,001)

The mean P wave duration for the lateral body position was 62,68 ms, for the dorsal body position the mean value was slightly lower and was 60,41 ms (*P*-value = 0,0346). The mean P wave amplitude value for the lateral body position was 0,1393 mV, while for the dorsal position it was 0,1055 mV (*P*-value = 0,0003). The mean PQ interval duration value for lateral body position was 114,7 ms, while for the dorsal position it was 108,9 ms (*P*-value = 0,0003). The mean R wave amplitude for the lateral body position was 0,2755 mV and for the dorsal body position, the mean value was 0,2190 mV (*P*-value = 0,0465). The mean QT Interval duration in lateral position was 341,3 ms, while for the dorsal position it was 335,5 ms (*P*-value = 0,0208) (Fig. 13). In lateral position biphasic P wave was observed in one specimen, negative deflection value was -0,04 mV. In dorsal position no biphasic P waves were observed. R’ wave was found in one specimen in dorsal body position—amplitude varied between 0,05 and 0,11 mV. In lateral body position R’ wave was also found in one animal—amplitude varied between 0,09 and 0,12 mV. Absence of R wave was observed in 6 animals in lateral position while in dorsal position R wave was not found in 7 animals.

### Lead V6

**Fig. 14:**
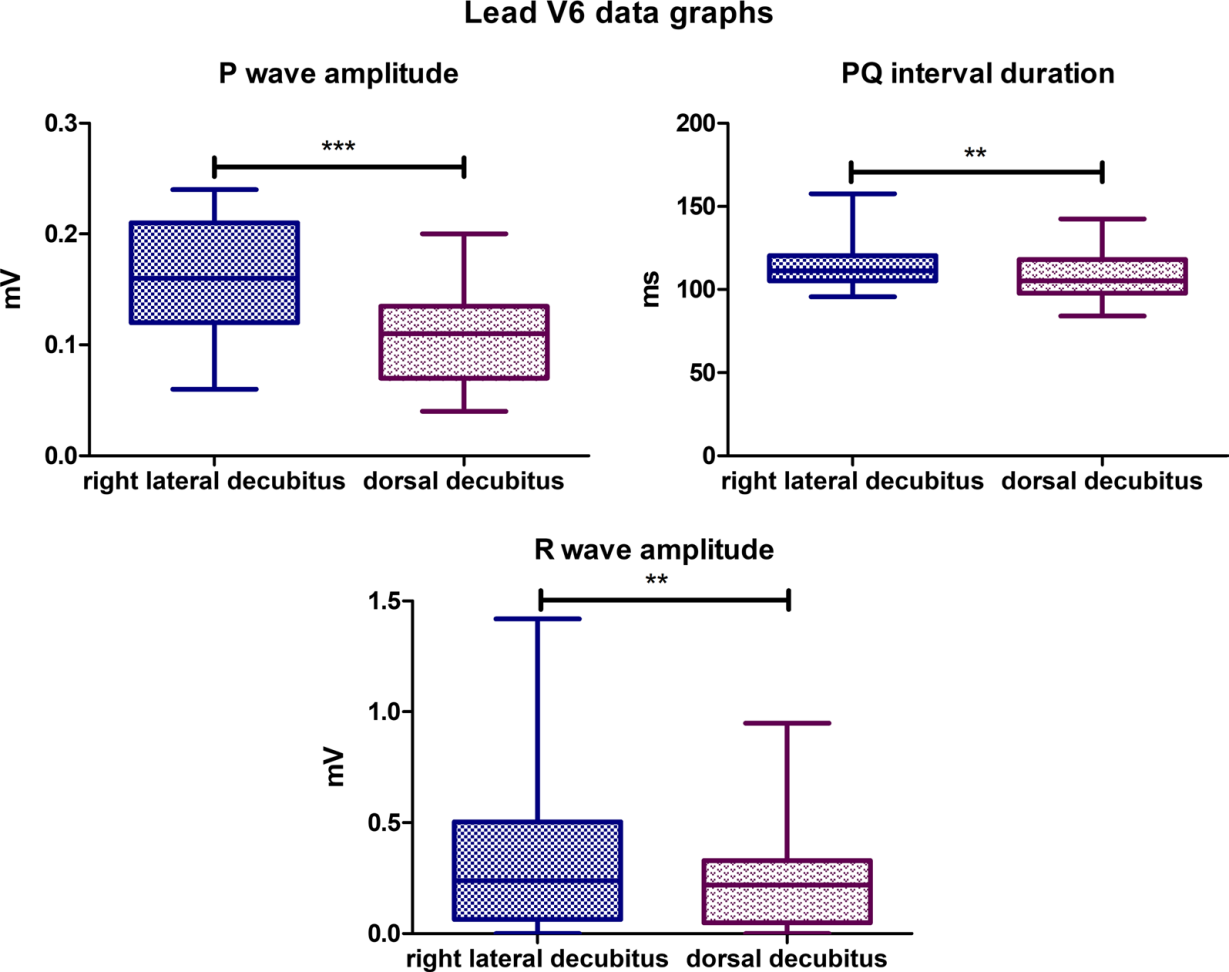
Comparison of the recording parameters differing statistically significantly in lead V6. T test for dependent pairs performed on 29 animals, where the values in the same specimen obtained in 2 body positions constitute a pair. Box plot contains 25% to 75% percentile, the inside line represents median, and whiskers represent minimal and maximal values (**P < 0,01, ***P < 0,001)

The mean P wave amplitude value for the lateral body position was 0,1576 mV, while for the dorsal position it was 0,1048 mV (*P*-value < 0,0001). The mean PQ interval duration value for lateral body position was 114,3 ms, while for the dorsal position it was 108,5 ms (*P*-value = 0,0013). The mean R wave amplitude for the lateral body position was 0,3231 mV and for the dorsal body position, the mean value was 0,2345 mV (*P*-value = 0,0046) (Fig. 14). A biphasic P wave was observed in the lateral position in 2 specimens with a negative deflection ranging from − 0,02 mV to − 0,03 mV. In the dorsal position, no biphasic *P* waves were observed in lead V6. R’ wave was observed in 1 specimen in dorsal body position, the amplitude = 0,05 mV. In lateral position, R’ wave was also observed in 1 animal—the amplitude varied between 0,12 and 0,14 mV. The absence of R wave was observed in 4 animals in lateral position while in dorsal body position, it was 9 specimens.

### Establishment of ECG normative values for right lateral and dorsal body position

Based on the obtained results, the normative values for the ECG recordings in swine for the right lateral and dorsal position were developed (Tables 1 and 2).

**Table 1:** Normative ranges for right lateral body position

Lead	P wave duration [ms]	P wave amplitude [mV]	PQ duration [ms]	QRS duration [ms]	R wave amplitude [mV]	QT interval duration [ms]	QTc [ms]
I	49,62–70,73	0,09–0,16	104,06–134,12	66,54–80,52	0,14–0,63	307,07–373,65	386,41–458,75
II	51,91–76,50	0,06–0,17	106,85–134,97	63,58–81,89	0,30–1,03	303,37–375,72	382,40–460,42
III	46,95–71,49	(− 0,06)–(0,07)	100,98–138,11	58,12–77,49	0,33–1,10	304,96–376,20	384,31–460,92
aVR	51,96–71,90	(− 0,16)–(− 0,06)	104,19–131,73	67,52–80,72	0,10–0,25	302,12–375,17	380,23–460,39
aVL	43,78–68,81	0,03–0,10	104,26–136,98	62,64–76,37	0,12–0,43	304,84–373,93	383,85–458,47
aVF	48,92–70,06	0,01–0,10	105,71–136,72	60,55–78,16	0,35–1,01	305,94–373,69	384,07–459,71
V1	50,28–69,61	(− 0,11)–(− 0,05)	104,82–132,69	60,97–76,11	0,23–0,52	313,27–375,38	392,52–462,80
V2	51,38–72,32	0,02–0,07	103,45–130,95	65,46–78,95	0,08–0,38	314,15–378,57	394,42–464,37
V3	52,01–73,99	0,04–0,11	103,41–129,67	63,69–78,96	0,07–0,41	312,53–377,51	391,79–465,06
V4	51,55–72,48	0,07–0,16	100,38–128,59	64,41–77,94	0,05–0,53	308,73–374,64	385,36–463,11
V5	52,86–72,49	0,09–0,19	101,37–128,01	63,21–79,90	0,04–0,61	308,60–373,91	385,61–462,13
V6	53,77–72,80	0,10–0,21	99,12–129,49	61,45–79,05	0,04–0,68	308,51–374,71	387,02–461,36

**Table 2:** Normative ranges for dorsal body position

Lead	P wave duration [ms]	P wave amplitude [mV]	PQ duration [ms]	QRS duration [ms]	R wave amplitude [mV]	QT interval duration [ms]	QTc [ms]
I	54,66–71,90	0,13–0,23	101,51–129,28	67,77–81,31	0,14–0,81	301,28–376,13	392,21–467,10
II	54,44–74,77	0,07–0,18	103,32–131,96	62,30–79,49	0,21–0,92	301,58–376,85	385,57–472,32
III	43,83–67,99	(− 0,10)–(0,04)	92,30–127,84	61,27–78,76	0,20–0,82	306,47–373,28	392,35–468,63
aVR	55,43–74,61	(− 0,19)–(− 0,11)	101,01–129,11	65,63–82,34	0,12–0,31	299,65–377,35	386,67–470,14
aVL	49,38–68,94	0,07–0,16	100,57–130,66	62,27–78,92	0,13–0,65	305,30–370,21	391,06–464,69
aVF	43,79–64,51	0,01–0,09	100,47–130,12	56,73–71,87	0,20–0,79	304,30–371,23	389,95–465,69
V1	52,29–69,56	(− 0,15)–(− 0,06)	98,98–130,45	62,35–77,01	0,23–0,58	306,84–375,20	393,45–470,84
V2	52,26–70,69	0,03–0,11	96,72–127,17	64,06–79,69	0,06–0,45	307,48–376,60	395,76–470,31
V3	52,06–70,56	0,05–0,14	96,71–125,64	60,48–78,64	0,02–0,46	302,70–373,47	388,81–467,45
V4	51,73–68,79	0,07–0,14	94,68–123,66	60,70–76,46	0,01–0,47	304,56–370,34	390,94–463,83
V5	52,71–68,11	0,07–0,14	94,98–122,75	62,29–75,97	0,03–0,50	302,72–368,37	388,23–461,78
V6	51,18–68,58	0,06–0,15	94,45–122,53	61,28–76,48	0,07–0,53	303,85–370,74	393,41–462,75

## Discussion

**Fig. 15:**
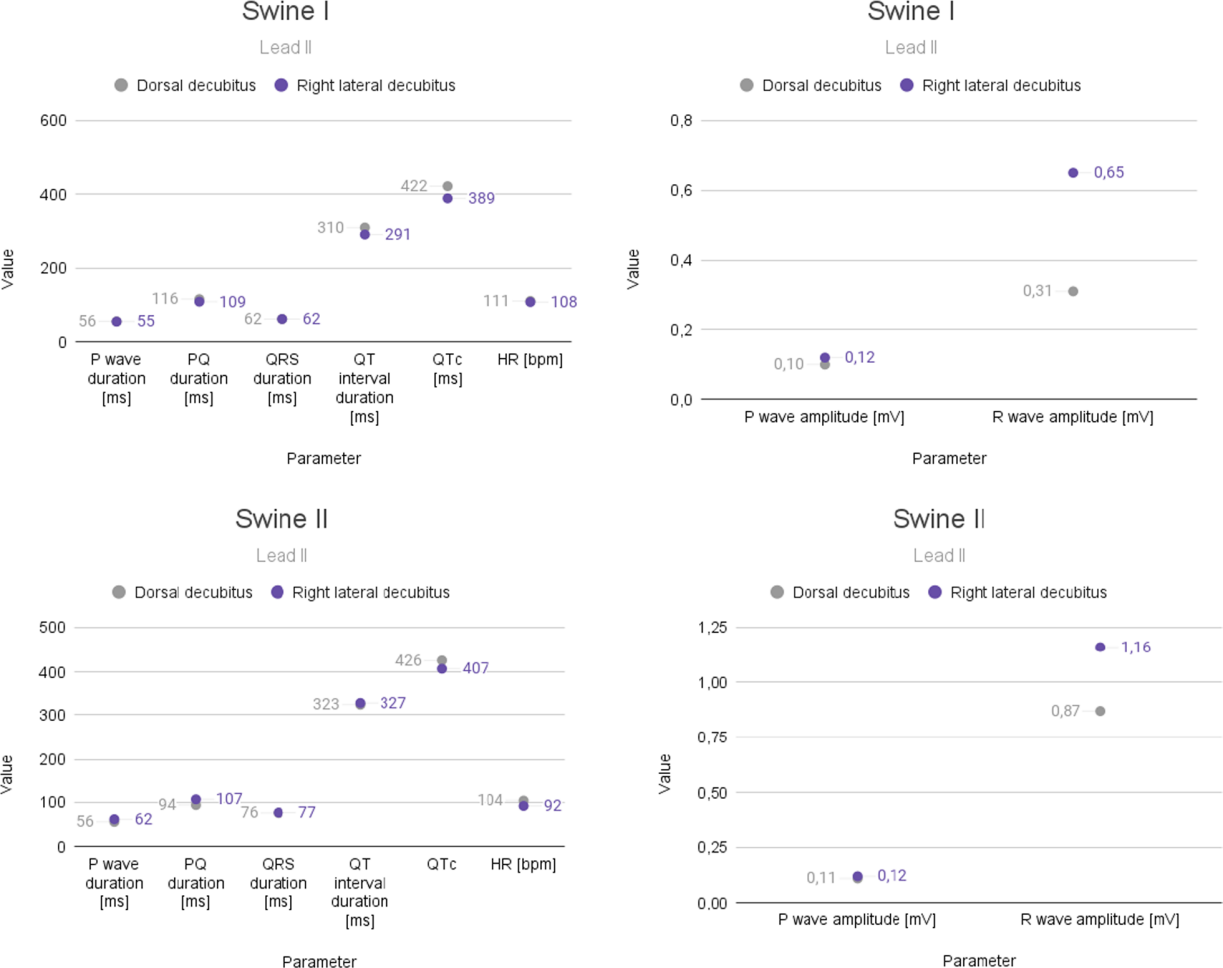
Comparison of ECG recording parameters in lead II in exemplary animals

**Fig. 16:**
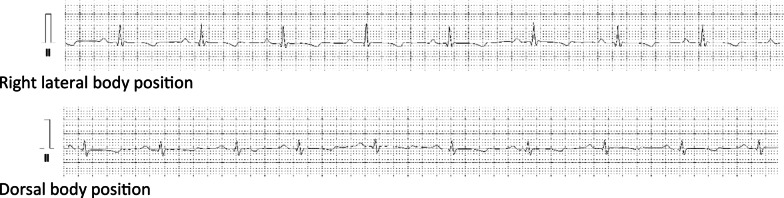
Comparison of fragments of the Lead II ECG recordings in an exemplary examined animal (50 mm/s; 10 mm/mV; 50 Hz notch filter; Fuzzy + software filter)

As we suspected, there are statistically significant differences in the studied ECG recordings, which clearly result from a change in body position. These differences mainly relate to the amplitude of the P and R waves and are even more apparent when comparing the electrocardiogram of the same individual (Figs. 15 and 16). It is difficult to keep a steady heart rhythm in a patient, especially when manipulating animals bodies during examinations. We suspect that this could be the reason for statistically significant differences in the heart rate. We also suspect that the change in the recording could have been also influenced by the change in the position of the electrodes caused by the shift of the skin during the change of the animal's position—this was also mentioned in Rishniw M, Porciello F article [28]. Additional factors that we suspect may have influenced the ECG recording are the times between tests performed in different positions and the action curve of anesthetic drugs affecting the cardiovascular system, which of course constitute some limitations of our study.

Compared to Rishniw M, Porciello F, we also observed similar changes in the electric axis of the heart [28]. Interestingly, the dorsal decubitus in pigs gives similar electric axis values to the standing position in dogs. It is worth noting that the amplitude of the recording in pigs differs from that in dogs, which suggests that the standards of ECG recording for dogs will not be applicable to companion pigs, which may be of significant use in animal clinics, where the only known standards are often those for dogs and cats [28]. However, it should be borne in mind that this study was performed in conscious animals [28].

As our study proves, the change of the animal's position is associated primarily with the change in the amplitude of the ECG recording. We suspect that, as in dogs, in pigs, the increased or decreased amplitude of the QRS complex in a given position may be a misleading indication of various cardiac disorders such as left ventricular hypertrophy [28].

Comparing results to our previous study was limited because only lead II was then examined and different time units were used [22]. Despite this, we noticed differences in the designated reference ranges for the amplitudes of the P and R waves, which clearly suggests that the standards set for a specific body position should be used. In the studied population, in lead II, pigs achieved higher R-wave amplitudes, and the range of P-wave amplitudes is slightly narrower. Moreover, the tested animals were characterized by a longer QT interval and a higher QTc parameter. We suspect that this may have been due to the use of a different anesthetic protocol and its cardiovascular effects. When it comes to comparing wave duration and intervals, due to the difference in the units used, it is difficult to assess the differences unequivocally. Due to the lack of data on the remaining leads, we were unable to perform a broader comparison of these data. Considering the differences, we encountered even though examining the same breed and weight range of animals, it is important in further research to take into account the effects of the drugs used, which may be responsible for certain changes. We believe that the different doses in anesthesia protocol we used are also responsible for slight abnormalities in echocardiographic parameters of shortening fraction, ejection fraction and E/A ratio.

Importantly, the electrocardiographic recording obtained with the applied precordial lead system recommended by Santilli et al. for dogs was of good, clear quality [29].

When comparing the results obtained by us to the tests performed in anesthetized dogs, significant differences can also be found [30, 31]. In dogs, in lead II, the ECG is characterized by higher heart rate, higher amplitude of P and R waves, shorter PQ and QT intervals, and shorter QRS complexes. Such observations indicate that it is not advisable to use ECG standards interchangeably between species. Certain limitations resulting from the influence of different anesthesia protocols in the compared studies should also be considered.

Comparing the parameters obtained by us with those from conscious Göttingen miniature pigs, we observed some significant differences [19, 20]. Polish Landrace Pigs are characterized by longer PQ and QT intervals and a longer QRS complex, even with a lower HR than in animals tested by Nahas K, Baneux P, Detweiler D [20]. Additionally, the pigs studied by us had different amplitudes of the P and R waves in individual leads. Interestingly, freely moving Göttingen minipigs in resting ECG were characterized by a lower HR than the animals tested by us [19].

In the case of anesthetic monitoring, it is the procedure that determines the position of the animal, while the anesthesiologist is responsible for the choice of ECG leads that will be used to analyze the patient's condition. The pigs examined by us showed a high variability of the ECG record between individuals, therefore we are not able to recommend a specific ECG lead for monitoring, we experienced the highest variability especially in the dorsal position.

When it comes to recommending the optimal position for ECG examination in pigs, it seems reasonable that the standard position of the resting ECG should be the physiological standing position. This position should be the least stressful for the conscious animal and give the most precise results. When it comes to examining an anesthetized animal, the right lateral decubitus seems to be the best choice. This is a position that is the golden standard in dogs [29]. Given our positive experience with the electrode placement system used in dogs and the similarity of the chest structure between these species, we believe that the use of the right lateral decubitus will provide results of high diagnostic value. However, the right lateral decubitus is not always achievable, for example in the case of right chest injuries, hence the dorsal position seems to be the second choice in this case for which reference values we also developed.

Due to the increasing popularity of pigs as companion animals, providing veterinary aid in this species will require getting increasingly more knowledge of this species as a potential patient in veterinary clinics [1, 2]. Also, recent spectacular achievements in the field of xenotransplantology and model research concerning, inter alia, heart and kidney transplants show how important it is to broaden knowledge about this species [9–12]. Increasingly advanced medical procedures are performed in swine, including orthopedic surgeries or minimally invasive cardiology procedures, both in companion pigs and during model tests [23, 24, 32]. Therefore, it is more and more important to carefully monitor the patient's condition during anesthesia, as in human medicine or during anesthesia of other companion animals. We strongly believe that the research we have performed should be extended in the future. As pets, miniature pigs are the most popular. Considering the large breed and weight of the animals we examined, we believe that further studies should be done on smaller breed pigs. In addition to applying the results of our study to procedures performed on anesthetized animals, our results may be a good introduction to further studies in conscious swine in a physiological posture. Performing the examination under general anesthesia and in such a weight, age and gender group is, of course, a limitation of the study, but we believe that our results can be applied and used in the future.

## Conclusions

The position of the body in domestic swine has a significant influence on the electrocardiogram. Due to the need for different patient positioning during the procedures performed wither in pigs as pets and in model studies, it is important to use ECG reference values appropriate for the standard position, which will allow for more effective evaluation of the recording and monitoring of the patient. The applied system of limb and precordial leads has been successfully used in pigs.

## Methods

The study was carried out on 29 female Polish landrace pigs weighing in the range of 33–44 kg of age between 16 and 20 weeks. All animals were purchased from one breeding farm. Each animal before procedures underwent a 14-day quarantine to prevent the influence of stress factors on the results. Animals were also generally examined, to ensure all were clinically healthy. Blood samples were collected for biochemistry, coagulology and morphology tests (Tables 3 and 4). The departmental laboratory's internal reference ranges were used, for the TT parameter ranges published by Olsen et al. were used [33]. Echocardiography was performed, all animals were within the reference ranges (Table 5) [22]. Only slight nonsignificant individual deviations from the norms were observed.

**Table 3:** Biochemistry and coagulology values

	AspAT [U/I]	AlAT [U/I]	AP [U/I]	Urea [mmol/l]	Creatinine [umol/l]	Total protein [g/l]	Albumin [g/l]	Total bilirubin [umol/l]	Total calcium [mmol/l]	Sodium [mmol/l]	Potassium [mmol/l]	Chlorides [mmol/l]	PT [s]	APTT [s]	TT [s]	Fibrinogen [g/l]
Mean	32,17	82,07	275,37	5,01	119,00	58,74	30,50	12,93	2,32	134,97	3,60	100,10	12,99	49,09	17,14	3,61
Median	31,00	81,00	275,00	5,00	117,00	57,00	30,00	13,30	2,30	135,60	3,62	100,60	12,60	44,00	10,85	3,47
Standard deviation	8,40	19,83	79,77	1,16	42,56	14,56	3,49	5,32	0,21	3,06	0,24	3,11	1,75	18,75	21,46	0,80
Reference range	16–65	9–43	92–294	3,3–6,6	88–239	59–74	31–50	0–10	2–4	139,1–156,5	4,4–5,6	95,8–110	11–13	15–21	23–35	2–4

*AspAT* aspartate transaminase; *AlAT* alanine transaminase; *AP* alkaline phosphatase; *PT* prothrombin time; *APTT* activated partial thromboplastin time; *TT* thrombin time

**Table 4:** Blood count values

	WBC [G/l]	RBC [T/l]	Hb [mmol/l]	HCT [l/l]	MCV [fl]	MCH [fmol]	MCHC [mmol/l]	PLT [G/l]
Mean	14,97	5,89	6,04	0,31	53,26	1,03	19,27	434,19
Median	13,20	5,85	6,10	0,31	53,0	1,03	19,30	450,00
Standard deviation	6,91	0,49	0,52	0,02	2,47	0,06	0,58	105,75
Reference range	10–20	5–8	6,21–9,93	0,32–0,5	50–68	1,05–1,43	18,6–21,08	120–450

*WBC* white blood count; *RBC* red blood cell count; *Hb* haemoglobin; *HCT* haematocrit; *MCV* mean corpuscular volume; *MCH* mean corpuscular hemoglobin; *MCHC* mean corpuscular hemoglobin concentration; *PLT* thrombocytes

**Table 5:** Echocardiographic values

	LA/Ao	IVSd [mm]	LVIDd [mm]	LVPWd [mm]	IVSs [mm]	LVIDs [mm]	LVPWs [mm]	EF [%]	FS [%]	HR [bpm]	RVOT [m/s]	LVOT [m/s]	eV [m/s]	aV [m/s]	E/A	TR Vmax [m/s]
Mean	1,38	8,31	40,54	6,58	11,85	26,04	11,77	65,04	35,72	100	0,92	1,24	0,74	0,65	1,22	2,85
Median	1,42	8,00	40,50	6,00	12,00	26,50	12,00	65,75	36,00	94	0,91	1,17	0,73	0,63	1,23	2,81
Standard deviation	0,16	1,38	5,14	1,45	2,09	4,11	1,50	9,00	7,53	22	0,11	0,29	0,12	0,18	0,36	0,31
Reference range	1,27–1,66	5,7–0,8,4	37,7–47,0	5,8–7,7	9,4–11,9	24,5–33,0	10,3–13,5	50–63	27–34	–	–	–	–	–	1,76–2,1	–

*LA/Ao* left atrial size-to-aortic root diameter ratio; *IVS* interventricular septum thickness, *LVID* left ventricular internal diameter, *LVPW* left posterior wall thickness, *FS* fractional shortening, *EF* ejection fraction; *HR* heart rate; *RVOT* right ventricular outflow tract; *LVOT* left ventricular outflow tract; *eV* early diastolic transmitral flow velocity; *aV* late (atrial) diastolic transmitral flow velocity; *TR* tricuspid regurgitation, *d* end-diastole; *s *end-systole

**Fig. 17:**
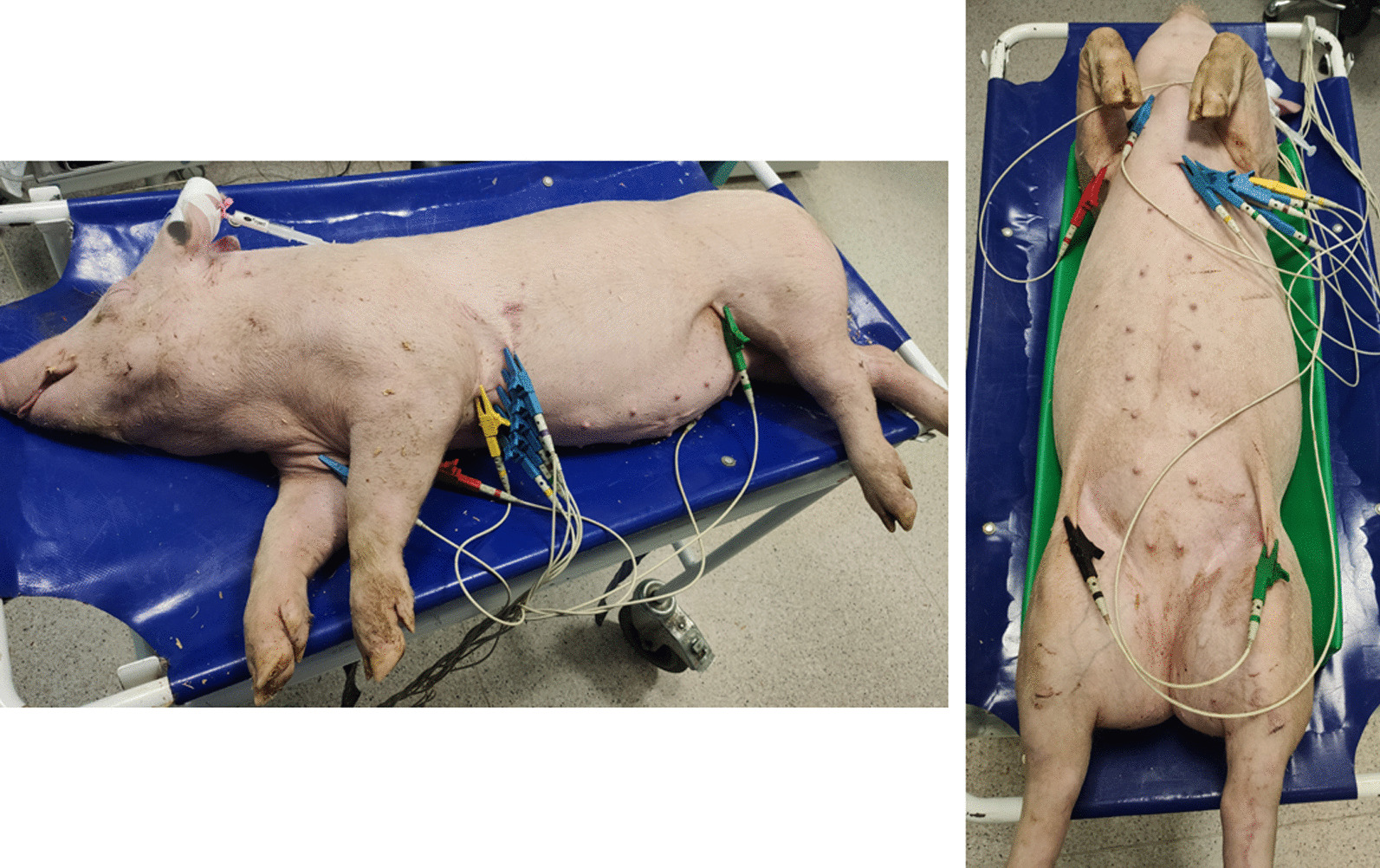
Presentation of animals positioning, and precordial and limb electrode placement system used in the study

The tests were performed under general anesthesia using a standardized protocol for each animal. The anesthesia protocol included an intramuscular injection of medetomidine 0,03 mg/kg (Sedator, Eurovet Animal Health BV, Netherlands), midazolam 0,3 mg/kg (Midanium, Polfa Warszawa S.A., Poland), ketamine 10 mg/kg (Vetaketam, Vet-Agro Sp. z.o.o., Poland), and intravenous injection of propofol 2 mg/kg (Provive, Claris Lifesciences UK Ltd., UK). ECG examination was performed in two positions: right lateral position and next on the back (Fig. 17). For assurance of a stable and repeatable position of the body on the back, a radiological positioner was used. Recordings were collected and analyzed using a 12-lead BTL-08 MT Plus resting ECG system with BTL-08 Win 6.14 software support (BTL Industries Ltd, UK). The following test parameters were applied: 50 mm/s; 10 mm/mV; 50 Hz notch filter; fuzzy + software filter. Due to the lack of literature data on the electrode placement system for swine, precordial electrodes were placed as recommended by Santilli et al. [13] for dogs with a similar chest structure. Limb electrodes were placed in a standard system [10].

Resting ECG records in both positions were made in every animal, and then 5 consecutive sinus stimulations were measured in each lead. ECGs were analyzed to determine such parameters as P wave duration and amplitude, PQ interval duration, QRS complex duration, R wave amplitude, QT interval duration, QTc value using Bazett formula, Heart rate (HR), Electrical heart axis, occurrence of biphasic P and its amplitude, occurrence of R’ wave and its amplitude, non-occurrence of R wave. The electrical heart axis was determined by using the I and III lead QRS net vector in the hexaxial reference system. Obtained results were statistically analyzed in the Graphpad Prism v5.03 program using a t-test for dependent pairs. On the basis of the results, normative values for ECG in dorsal and right lateral position were developed using the mean ± sd formula. Due to the definition of R wave, which cannot be negative, in some cases, the formula was changed, and the lower value of the norm was set to zero. The study was approved by the Local Ethical Committee for Animal Experiments in Wrocław, Hirszfeld Institute of Immunology and Experimental Therapy—approval nr 081/2019.

## Data Availability

The datasets used and/or analysed during the current study are available from the corresponding author on reasonable request.
